# Explainable Machine Learning for Heat-Related Illness Prediction: An XGBoost–SHAP Approach Using Korean Meteorological Data

**DOI:** 10.3390/bioengineering12111276

**Published:** 2025-11-20

**Authors:** Chaeyeong Im, Wonji Kim, Heesoo Kim

**Affiliations:** 1 The Armed Forces Medical Command, Ministry of National Defense, Seongnam 13574, Gyeonggi-Do, Republic of Korea; idisdanielcy@gmail.com; 2 Department of Pediatric Oncology, National Cancer Center, Goyang 10408, Gyeonggi-Do, Republic of Korea; wonjk67@gmail.com; 3 Department of Artificial Intelligence Convergence, Gwangju Institute of Science and Technology (GIST), Gwangju 61005, Republic of Korea; 4 Department of Artificial Intelligence, Chonnam National University, Gwangju 61186, Republic of Korea

**Keywords:** heat-related illness, machine learning, explainable artificial intelligence, Shapley additive explanations, climate change, meteorological data

## Abstract

The rising frequency of heat-related illnesses (HRIs) under climate change presents urgent public health challenges, particularly in urban environments. This study develops an explainable machine learning (ML) model to predict HRI risk using metrological data from seven major South Korean metropolitan cities between May and September 2021–2024. We applied eXtreme Gradient Boosting (XGBoost) to model relationships between daily meteorological variables, including maximum and mean daily temperatures, humidity, solar radiation, wind speed, and precipitation, and HRI occurrence. Model performance was validated using 2025 data and demonstrated strong predictive accuracy, with area under the curve (AUC) values 0.895. To enhance interpretability, Shapley Additive exPlanations (SHAP) analysis identified mean daily temperature, solar radiation, and minimum temperature as the strongest contributors to HRI risk. Time-series comparisons of predicted and actual HRI occurrences further validated the model’s effectiveness in real-world settings. These findings underscore the potential of eXplainable Artificial Intelligence (XAI) for localized health-risk forecasting and support a data-driven basis for developing early warning systems for climate-sensitive diseases to guide proactive public health planning amid escalating urban heat risks.

## 1. Introduction

Climate change has intensified the frequency, severity, and duration of extreme heat events globally, posing significant public health risks, particularly in urban settings. Heat-related illnesses (HRIs), ranging from mild conditions such as heat exhaustion and cramps to more severe outcomes such as heat syncope, heatstroke, and multi-organ failure, are increasingly prevalent among vulnerable populations, including the elderly, outdoor workers, and those with chronic diseases. Without timely intervention, HRIs may lead to irreversible neurological damage or death, underscoring the importance of early detection and prevention [1,2].

In South Korea, heatwave impacts have intensified in recent years, with the Korea Disease Control and Prevention Agency (KDCA) reporting a sharp increase in both HRI cases and the severity of clinical outcomes, including hospitalizations and heatstroke-related deaths, during peak summer months. This trend reflects the urgent need for proactive public health strategies to address climate change-driven heat risks [1,2].

Timely prediction of HRIs is critical for public health planning, early warning systems, and targeted interventions. While conventional statistical approaches, including logistic regression and time-series models, estimate heat-related health risks, these models often struggle to capture complex nonlinear interactions among meteorological variables [3,4,5,6,7,8,9,10,11]. Recent advancements in machine learning (ML) offer improved predictive performance and the capacity to process high-dimensional data [12,13,14,15,16,17]. However, several ML models remain limited by their “black-box” characteristics, hindering clinical or policy-level adoption due to insufficient transparency and interpretability [18,19].

To overcome these challenges, eXplainable Artificial Intelligence (XAI) techniques, such as Shapley Additive Explanations (SHAP), have been developed to examine the contribution of individual features to model predictions [18,19]. Such approaches are particularly critical in public health, where interpretability of the prediction is as important as predictive accuracy [6,7,8].

This study developed a weather-driven HRI prediction model using the eXtreme Gradient Boosting (XGBoost) algorithm, applied to meteorological data collected from seven major South Korean cities between 2021 and 2025 [20,21]. To enhance interpretability, SHAP analysis was incorporated to determine influential variables and visualize their effects [18,19]. Our findings aim to support data-driven, real-time public health monitoring and support the development of early warning systems tailored to climate-stressed urban environments [1].

## 2. Related Work

HRIs encompass a spectrum of clinical conditions caused by prolonged exposure to high temperatures, often exacerbated by high humidity. These include heat cramps, exhaustion, syncope, and the most severe form, heatstroke, which can cause organ failure and death if untreated [2] (Table 1). HRIs are particularly prevalent during summer and disproportionately affect vulnerable groups such as the elderly, outdoor workers, individuals with pre-existing conditions, and residents of urban heat islands [2,5]. The increasing intensity and frequency of heatwaves driven by climate change have amplified the global burden of HRIs, including in South Korea [1,6,7].

**Figure 1:**
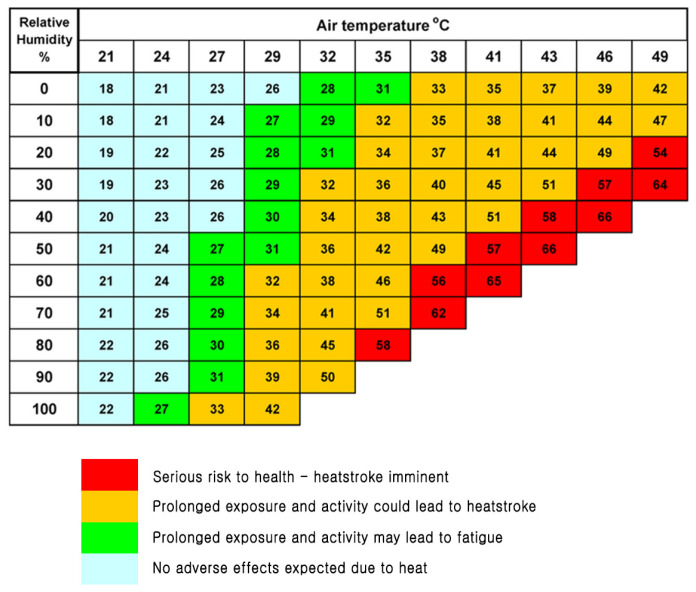
Heat Index chart (Apparent temperature).

The Heat Index (HI) is widely used to quantify heat stress. It integrates ambient temperature and relative humidity to reflect the perceived temperature experienced by the human body (Figure 1) [4]. HI > 27–30 °C may cause mild symptoms such as fatigue and dizziness, while HI > 40 °C significantly increases the risk of heat exhaustion or heatstroke [4]. Globally, HI serves as a key indicator for issuing heat advisories and guiding reference thresholds for public health alerts [4].

Previous studies have explored the relationship between environmental variables and HRIs using linear, logistic, and time-series regression models [8,9,10,11]. Although effective to some extent, these techniques struggle to capture complex, nonlinear interactions among climatic variables, limiting their predictive power. Recent studies have employed ML techniques, such as random forests (RFs) and support vector machines (SVMs), to enhance performance [12,13,14,15,16,17]. However, the limited transparency of these models continues to hinder their trust and adoption by clinicians and policymakers in real-world settings [18,19].

To bridge this gap, our study leverages XGBoost for its robust predictive capabilities and integrates SHAP to enhance model interpretability [18,19,20]. This integrated approach enables an accurate forecast of HRI risk while revealing the key environmental contributors to heat-related illnesses across diverse urban regions [6,7].

## 3. Materials and Methods

### 3.1. Dataset

#### 3.1.1. Data Preprocessing

This ecological, city-level study utilized publicly available meteorological and health surveillance data collected in South Korea during the summer (May–September) of 2021–2025. The analysis encompassed seven major metropolitan areas: Seoul, Busan, Daegu, Daejeon, Gwangju, Ulsan, and Incheon.

The training dataset comprised 3752 daily city-level observations (one observation per city per day) from May 20 to September 30 between 2021 and 2024, aggregated over a 4-year period. An additional 546 observations from May 15 to 31 July 2025, were reserved as a temporal test set to evaluate the model’s generalizability in real-world forecasting scenarios. The 2025 dataset was strictly held out for temporal validation only and was never used in model training, tuning, or oversampling procedures.

**Figure 2:**
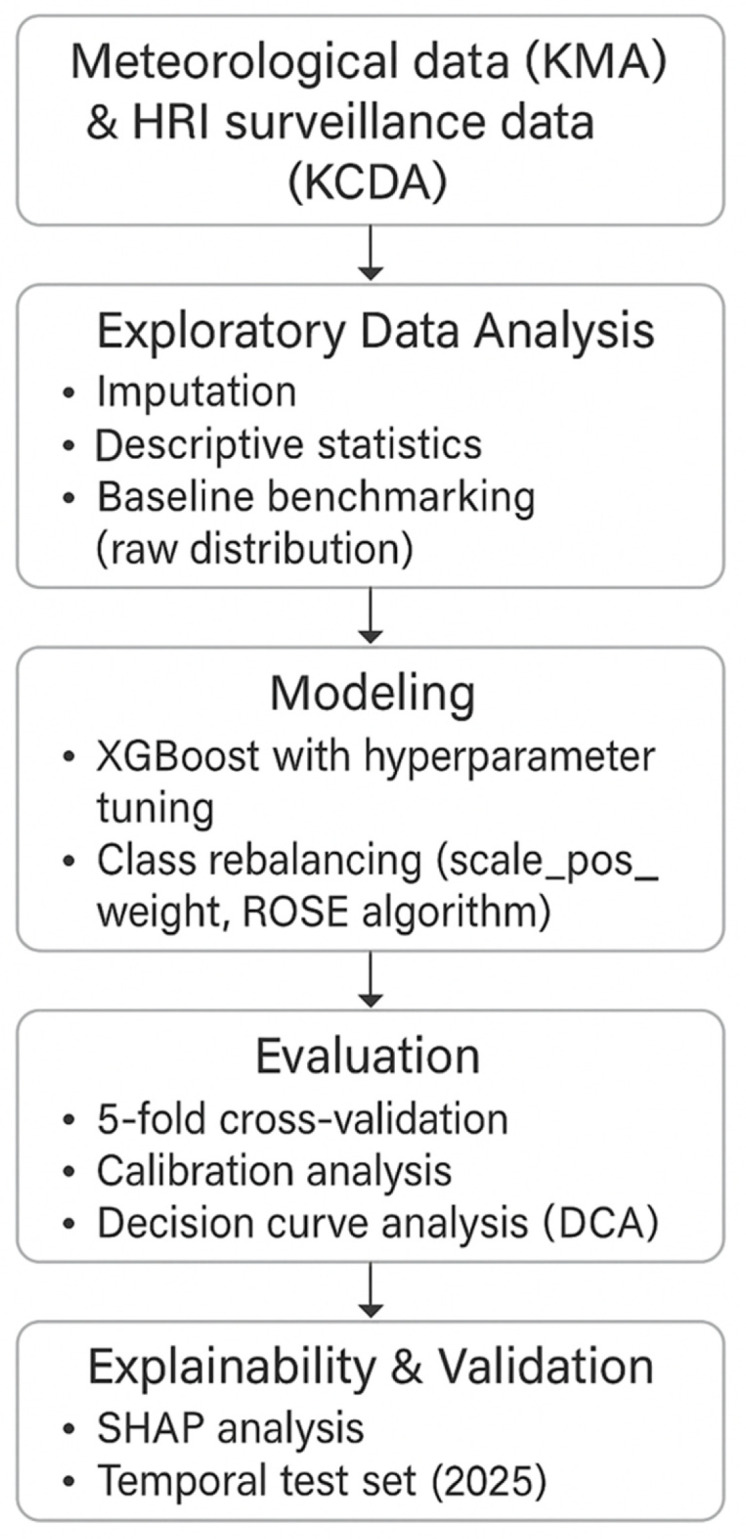
Overall framework of HRI prediction integrating meteorological and surveillance data.

HRI data were obtained from the Korea Disease Control and Prevention Agency’s (KDCA) National HRI Surveillance System, which records city-level emergency department visits for heat-related conditions [7,22]. Meteorological variables, including temperature, humidity, wind speed, solar radiation, and precipitation, were sourced from the Korea Meteorological Administration’s (KMA) Open Weather Portal [3,23] (Figure 2). All records were aggregated at the daily city level, with no individual-level information included. Missing or invalid values in core variables were imputed before model training.

Missing values were imputed using rule-based domain knowledge:

- Precipitation: Missing entries were assumed to indicate no rainfall and imputed as 0 mm, following standard meteorological practices [3].
- Wind speed, humidity, and solar radiation: Missing values were substituted with the overall training-period mean to prevent bias or artificial variance.

All data had undergone prior data quality control by the KMA [3]. Therefore, no additional outlier removal was required. All continuous predictors were standardized (z-score normalization). Sensitivity analysis comparing model AUCs before and after imputation showed negligible change (<0.01), confirming that the imputation procedure did not bias model performance.

#### 3.1.2. Definition of Variables

The dependent variable was a binary indicator representing the presence (1) or absence (0) of at least one reported HRI case per city per day. This information was derived from the KDCA’s National HRI Surveillance System, which reports daily city-level emergency department visits related to heat exposure [7,22].

Nine meteorological variables included as predictors, chosen for their physiological relevance to heat stress and supported by prior studies [4,5,8,9], were mean daily temperature (°C), maximum temperature (°C), minimum temperature (°C), temperature range (°C), mean daily relative humidity (%), minimum relative humidity (%), precipitation (mm), mean daily wind speed (m/s), and solar radiation (MJ/m^2^). All variables were recorded at daily intervals for each city using standardized automatic weather stations operated by the Korea Meteorological Administration and summarized in Table 2, including their names, abbreviations, units, and definitions [3,23].

### 3.2. Exploratory Analysis and Baseline Benchmarking

#### 3.2.1. Exploratory Data Analysis (EDA)

Prior to model training, exploratory data analysis (EDA) was conducted to examine the distributions of meteorological variables and their associations with daily HRI occurrence. Pearson correlation analysis evaluated linear relationships among numeric variables, while heatmaps visualized pairwise correlation coefficients. Scatterplots and boxplots were constructed to examine variable distributions and potential threshold effects for selected predictors. The analysis emphasized temperature-related features (e.g., mean daily and maximum temperature), humidity, and solar radiation, including their nonlinear interactions with HRI incidence. EDA was conducted using R (version 4.5.1) packages [21].

#### 3.2.2. Baseline Benchmarking

To benchmark performance under the natural class distribution, five baseline classifiers were implemented: (1) logistic regression, (2) RFs, (3) SVM, (4) k-nearest neighbors (k-NN), and (5) XGBoost. All models were trained on the original, unbalanced training dataset to compare predictive performance under identical data conditions, before applying any resampling or weighting adjustments.

A five-fold cross-validation (trainControl(method = “cv”, number = 5)) was performed using the area under the ROC curve (AUC) as the primary evaluation metric [10,11]. Predicted class probabilities from all folds were retained for post hoc ROC and calibration analyses. For non-tree models (logistic regression, SVM, k-NN), predictors were z-standardized within each fold, while tree-based models (RF and XGBoost) were trained on raw features.

Among the baseline algorithms, XGBoost achieved a competitive and strong discriminative performance and stable cross-validation results, comparable to logistic regression. Given its robustness and scalability, XGBoost was selected as the primary predictive framework for subsequent algorithmic enhancement [18,19,20]. To further improve sensitivity for the minority class, class rebalancing methods, including cost-sensitive weighting and synthetic oversampling via the Random Over-Sampling Examples (ROSE) algorithm, were applied during XGBoost optimization. The ROSE-rebalanced XGBoost achieved superior recall while maintaining comparable AUC and was thus adopted as the final predictive model for all downstream analyses (Section 3.3.2 and Section 3.4) [24].

### 3.3. Model Construction and Enhancement

#### 3.3.1. Balancing Data

The dataset exhibited a substantial class imbalance (Table 2), with most daily observations indicating no HRI (HRI = 0) and a smaller proportion representing days with at least one reported HRI case (HRI = 1). Such an imbalance can bias ML models toward the majority class, reducing sensitivity in detecting true positive HRI days.

To address class imbalance, two complementary rebalancing strategies were applied during XGBoost training:

- Cost-sensitive learning using the *scale_pos_weight* parameter, which adjusts the loss function to penalize misclassification of minority (positive) cases. The parameter was set to 2.8, reflecting the negative-to-positive HRI sample ratio (2762:990) in the training dataset.
- Synthetic oversampling using the ROSE algorithm, which generates balanced synthetic samples via smoothed bootstrapping.

Both approaches improved model recall and yielded a better trade-off between sensitivity and specificity relative to the original unbalanced model. The final XGBoost model was selected based on a sensitivity-focused evaluation, with the ROSE-based variant achieving slightly higher recall while maintaining comparable AUC.

This rebalancing step mitigated under-detection of HRI days and enhanced the model’s practical applicability for public health surveillance. The results (Table 3, Table 4, Table 5 and Table 6) demonstrate that appropriate class rebalancing substantially improves sensitivity for rare but critical heat-related events [12,13,15,16,17,18]. Unless otherwise noted, all subsequent analyses, including hyperparameter optimization and SHAP interpretation, were performed using the ROSE-balanced XGBoost model.

#### 3.3.2. Algorithmic Enhancement

To enhance predictive performance and stability, the ROSE-balanced XGBoost classifier was adopted as the final model. Class imbalance in the training dataset (HRI positive = 26.3%) was mitigated using the ROSE technique prior to model fitting.

Model training involved hyperparameter optimization conducted via grid search with five-fold cross-validation. Key parameters adjusted included *max_depth* (3–10), *n_estimators* (100–500), *learning_rate* (0.01–0.3), *subsample* (0.6–1.0), and *colsample_bytree* (0.6–1.0). The final configuration, *max_depth* = 6, *n_estimators* = 300, *learning_rate* = 0.05, *subsample* = 0.8, and *colsample_bytree* = 0.8, was selected for optimal AUC and stable log-loss convergence, ensuring robust learning without overfitting.

Model reproducibility was ensured by fixing the random seed (123) and using identical cross-validation folds across experiments. The final model, XGBoost (ROSE), was subsequently evaluated on the independent 2025 test set (n = 546) to assess temporal generalizability. Compared with the baseline XGBoost model without rebalancing (AUC = 0.900, sensitivity = 0.681 on the 2025 test set), the ROSE-balanced variant achieved comparable discrimination (AUC = 0.895) but markedly improved sensitivity (0.771), reflecting a stronger ability to identify HRI-positive cases.

Although the overall AUC difference was marginal, the gain in sensitivity is particularly valuable from a public health and clinical perspective, where missing potential HRI cases poses a far greater risk than generating false positives. The ROSE-balanced model therefore provides superior clinical utility, enabling earlier detection and prevention of heat-related emergencies in vulnerable populations. These results confirm that ROSE rebalancing enhances temporal robustness and HRI detection without compromising overall discrimination, establishing a reliable framework for HRI prediction.

### 3.4. Feature Importance Analysis

#### 3.4.1. Explainable Artificial Intelligence (XAI)

Traditional ML models often function as “black boxes,” limiting the interpretability of predictions. This lack of transparency can hinder trust, particularly in public health applications where interpretability is essential for policy decisions and risk communication.

To address this limitation, XAI methods have been developed. XAI provides insights into how ML models generate predictions [18,19]. By enhancing model transparency and accountability, XAI enables clinicians, public health officials, and decision-makers to understand the factors driving risk predictions.

#### 3.4.2. Shapley Additive exPlanations (SHAP)

To enhance the interpretability of the XGBoost model, SHAP was applied as a unified framework to explain model predictions based on cooperative game theory. SHAP quantifies each feature’s marginal contribution to prediction outcomes by evaluating all possible feature combinations, offering both global and local interpretability.

This study employed the TreeSHAP algorithm, an optimized SHAP implementation for tree-based models to compute exact Shapley values for the XGBoost classifier trained on 2021–2024 meteorological data and validated on the 2025 test set. This approach enabled robust interpretation of model behavior under real-world conditions.

SHAP analysis quantified global feature importance by ranking variables according to their mean absolute SHAP values, identifying the dominant contributors to HRI risk. It further visualized feature effects and interactions through summary and dependence plots, revealing nonlinear relationships and threshold behaviors, such as sharp risk escalation near 24–25 °C mean daily temperature and ~20 MJ/m^2^ solar radiation. These analyses of SHAP enhanced model transparency and interpretability, facilitating clear result communication to stakeholders and supporting practical integration into public health early-warning systems.

Together, SHAP-based analyses provided an interpretable, quantitative framework for understanding the influence of meteorological variables on HRI occurrence and for validating the model’s reliability in real-world deployment.

### 3.5. Performance Evaluations

Model performance was evaluated using multiple complementary metrics, including AUC, accuracy, sensitivity (recall), specificity, precision, and F1-score [10,11]. In addition, the Brier score and calibration analysis assessed the agreement between predicted and observed probabilities, while decision curve analysis (DCA) quantified the model’s clinical and public health utility across varying probability thresholds [25].

To better reflect operational use in early warning systems, two classification thresholds were considered instead of the conventional cutoff of 0.5: (1) the Youden index, maximizing the sum of sensitivity and specificity, and (2) a sensitivity-prioritized threshold ensuring recall ≥ 0.75, determined empirically from validation results to minimize missed HRI events. This dual-threshold approach enables flexible adaptation depending on acceptable trade-offs between false positives and false negatives.

Model performance was evaluated on the 2025 temporal test set, serving as an external dataset to simulate real-world forecasting conditions. Confusion matrices and ROC curves detailed classification outcomes and discriminative performance, while calibration and decision curves offered complementary insights into probability accuracy and net decision benefit. All analyses, including data preprocessing, model training, threshold tuning, and visualization, were conducted using R (version 4.5.1) [21].

## 4. Results

### 4.1. Baseline Characteristics and Correlation Analysis

This study analyzed 4298 daily city-level observations across seven South Korean metropolitan areas: Seoul, Busan, Daegu, Daejeon, Gwangju, Ulsan, and Incheon, during the summer seasons (May–September) from 2021 to 2025. Of these, 3752 observations collected from 2021 to 2024 were used for model training, while 546 observations from 2025 were reserved for temporal validation to assess model generalizability under real-world forecasting conditions.

Descriptive statistics were computed to characterize the meteorological variables included in the analysis (Table 4). Across the seven metropolitan areas, mean summer temperatures ranged from ~23.9 °C to 25.1 °C, relative humidity from 70.7% to 80.9%, and solar radiation from 17.0 to 18.9 MJ/m^2^.

Prior to one-way Analysis of Variance (ANOVA), the Shapiro–Wilk test was applied to verify the normality of residuals, and the Levene test was used to assess the homogeneity of variances across cities. Although these variables exhibited broadly comparable climatic ranges, ANOVA results revealed significant between-city differences for all three factors (*p* < 0.001), indicating that mean temperature, humidity, and solar radiation levels varied notably among cities despite similar overall distributions.

**Figure 3:**
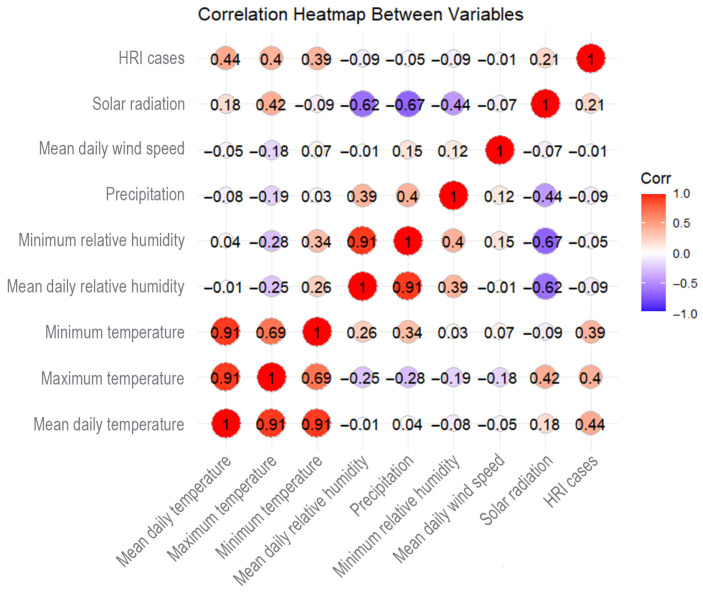
Correlation heatmap between meteorological variables, HRI cases.

Pearson correlation coefficients were calculated to assess relationships between predictors, with values ≥ 0.6 considered strong. The analysis revealed a very high correlation between maximum and mean daily temperatures (r = 0.91), as well as between minimum temperature and mean daily temperatures (r = 0.91). In addition, mean daily and minimum humidity showed a very strong correlation (r = 0.91). A moderately strong correlation was also observed between mean daily temperature and solar radiation (r = 0.42), indicating some redundancy among thermal and humidity indicators (Figure 3). Despite these results, no variables were excluded; however, potential multi-collinearity was carefully monitored during model fitting.

In terms of the relationship between meteorological variables and HRI cases, mean daily temperature demonstrated a moderate positive correlation with HRI cases (r = 0.44), while solar radiation exhibited a weaker but non-negligible positive correlation (r = 0.21) (Figure 3).

**Figure 4:**
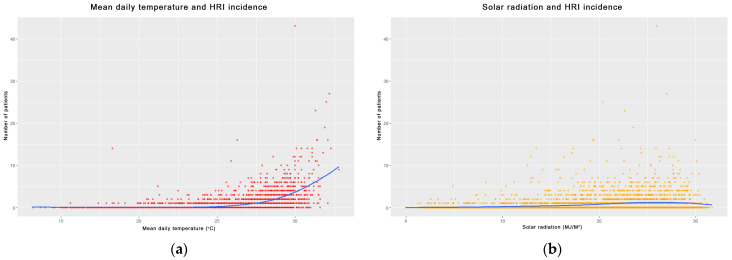
Scatter plots showing the relationships between meteorological variables and the number of HRI cases: (**a**) mean daily temperature and (**b**) daily solar radiation.

Scatterplots further highlighted nonlinear relationships between environmental exposures and HRI incidence. As depicted in Figure 4a, the number of HRI cases remained low until mean daily temperatures exceeded ~28 °C, after which incidence rose sharply. This threshold-like pattern suggests a nonlinear response of HRI risk to thermal exposure, later confirmed by generalized additive model (GAM) and SHAP analyses (Section 4.3). Similarly, solar radiation demonstrated a nonlinear relationship with HRI cases. In Figure 4b, the number of patients increased gradually with rising solar radiation, with a notable increase above 20 MJ/m^2^. This pattern underscores the relevance of solar exposure in HRI prediction models.

Table 5 summarizes the city-level descriptive statistics of daily HRI cases across the seven metropolitan areas to examine spatial variability. While absolute case counts differed, mean daily incidence per 100,000 population ranged narrowly from 0.0166 (Seoul) to 0.0514 (Ulsan), indicating comparable population-standardized HRI burdens nationwide. Although Seoul showed the highest raw mean and variability (1.55 ± 3.24), these differences diminished after population size adjustment. A nonparametric Kruskal–Wallis test revealed no statistically significant intercity differences in population-adjusted HRI rates (*p* = 0.423), suggesting that regional variations in absolute counts were primarily due to population density rather than disproportionate heat exposure risk. These findings support a unified national predictive framework while recognizing potential local heterogeneity in exposure-response dynamics.

### 4.2. Heat-Related Illness (HRI) Classification Performance

The binary classification task aimed to predict the occurrence of at least one HRI case per city per day. Five ML algorithms: logistic regression, RF, SVM, k-NN, and XGBoost, were trained and compared using five-fold cross-validation on the 2021–2024 dataset. The outcome variable was coded as 1 for HRI occurrence and 0 otherwise.

Model performance was evaluated using six metrics: AUC, accuracy, sensitivity (recall), specificity, precision, and F1-score. AUC served as the primary measure of discriminative ability, whereas sensitivity and F1-score were prioritized to minimize missed HRI events in public health forecasting [10,11,20,21].

To address class imbalance between HRI and non-HRI days, two rebalancing strategies were applied during further XGBoost training:

- Cost-sensitive weighting using the *scale_pos_weight* parameter;
- Synthetic oversampling using the ROSE algorithm.

These strategies substantially improved recall and F1-score, boosting F1-score from 0.626 to 0.779, without compromising AUC. This improvement demonstrates that balanced data representation enhances the model’s capacity to detect rare HRI events while maintaining overall discriminative performance.

**Figure 5:**
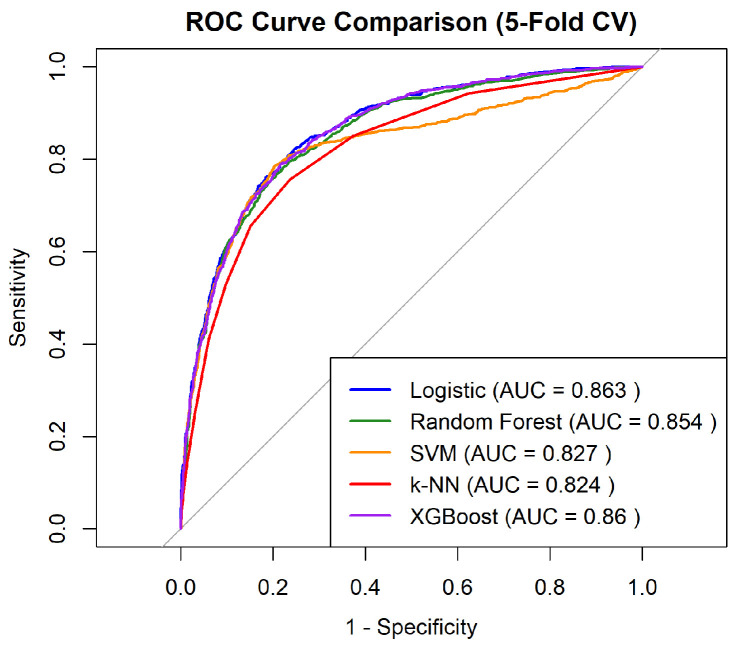
ROC curves of baseline benchmarking classification models (Logistic regression, RFs, SVM, k-NN, and XGBoost) trained on the unbalanced dataset (2021–2024).

Table 6 presents the comparative performance of all classifiers. While logistic regression yielded the highest AUC (0.863) at baseline benchmarking classification model (Figure 5), XGBoost demonstrated the most balanced performance across metrics, reaching 0.778 accuracy, 0.788 sensitivity, 0.768 specificity, and 0.779 F1-score after ROSE-based class rebalancing. This pattern reflects XGBoost’s robustness in capturing nonlinear feature interactions and seasonal variability, whereas simpler models such as SVM and k-NN showed limited sensitivity to rare positive cases. Consequently, XGBoost was selected as the final model for temporal validation and SHAP-based interpretability analysis.

**Figure 6:**
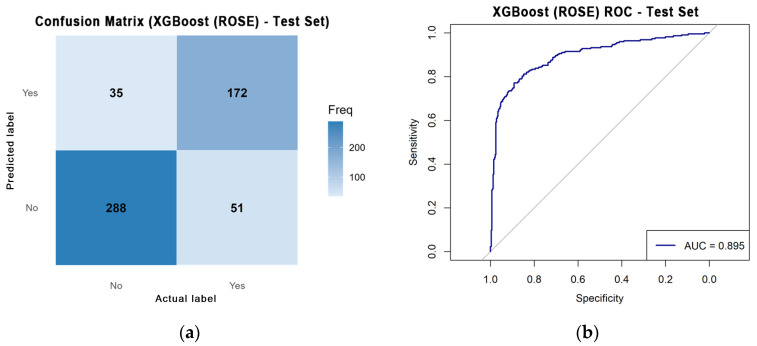
(**a**) Confusion matrix of the XGBoost (ROSE) model for predicting HRI occurrence using test data. (**b**) ROC curve of the XGBoost (ROSE) model on test data for predicting HRI occurrence.

To validate temporal generalizability, the XGBoost (ROSE) model was evaluated on independent 2025 data. The confusion matrix (Figure 6a) indicated high specificity (89.2%) and moderate sensitivity (77.1%), with an overall accuracy of 84.3%. Among 546 daily observations, 288 non-HRI days and 172 HRI days were correctly classified, with 51 false negatives and 35 false positives. The ROC curve yielded an AUC of 0.895, confirming strong discriminative performance even under real-world imbalance conditions (Figure 6b). The 95% confidence intervals were estimated using the normal approximation to the binomial distribution, yielding AUC 0.895 (95% CI 0.874–0.916), sensitivity 0.771 (95% CI 0.732–0.810), and specificity 0.892 (95% CI 0.858–0.923). Furthermore, the Brier score (0.1261) indicated good calibration between predicted probabilities and observed outcomes, supporting the model’s suitability for operational use in early warning systems [10,11].

These temporal validation results demonstrate that the proposed XGBoost (ROSE) framework maintains strong discriminative and calibration performance when applied to unseen 2025 data, confirming its robustness and reliability for real-world heat-risk surveillance.

### 4.3. Calibration and Explainability Analysis

**Figure 7:**
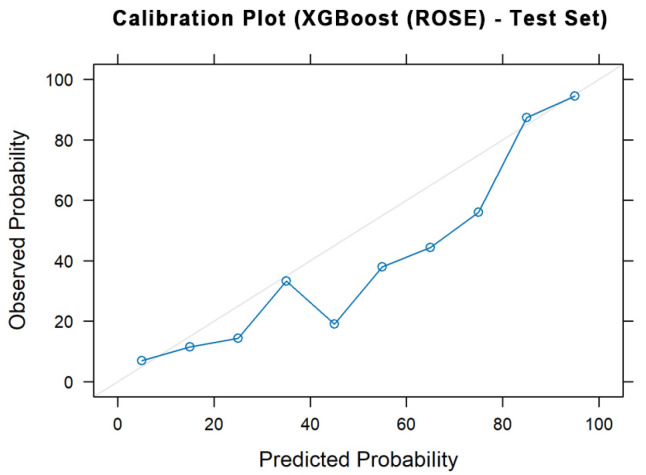
Calibration plot of the XGBoost (ROSE) model on test data.

Model calibration and interpretability analyses were conducted to determine the reliability and transparency of XGBoost predictions. The calibration curve for the 2025 test data (Figure 7) exhibited good alignment between predicted and observed probabilities, with a Brier score of 0.1261, indicating well-calibrated probability estimates. The curve closely followed the 45° reference line at higher probabilities, suggesting that the model’s output can be meaningfully interpreted as the likelihood of HRI occurrence in real-world conditions.

**Figure 8:**
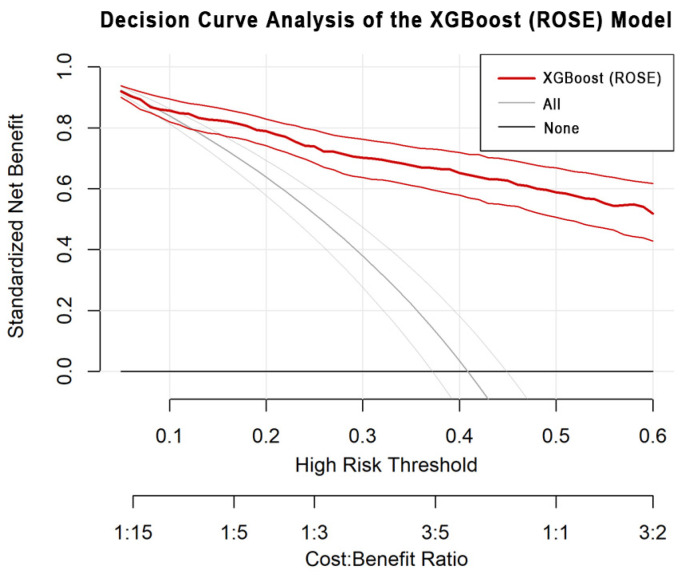
DCA results comparing the net benefit of the XGBoost (ROSE) model with ‘treat-all’ and ‘treat-none’ strategies.

To further assess the model’s clinical and operational utility, DCA was conducted (Figure 8). The DCA revealed that the XGBoost (ROSE) model consistently provided higher standardized net benefit across a broad range of high-risk thresholds (0.1–0.5) compared with “treat-all” or “treat-none” strategies. This result suggests that the proposed model yields a superior trade-off between true-positive detection and false-positive costs, reinforcing its potential value for public health early warning systems.

**Figure 9:**
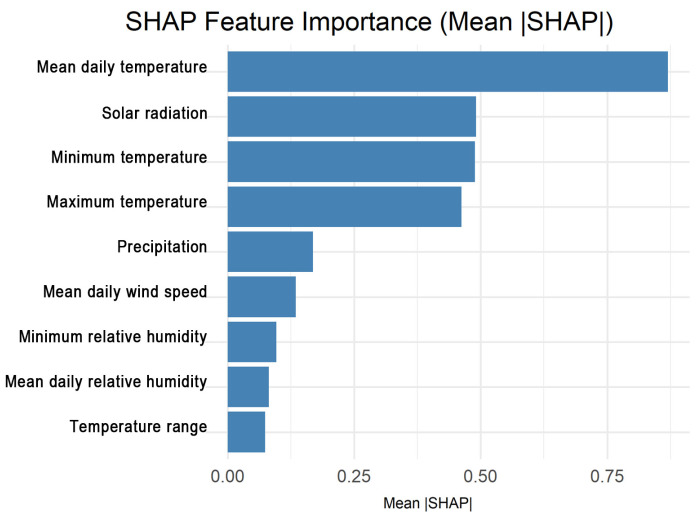
SHAP feature importance ranking (mean |SHAP| values) showing the top meteorological predictors of HRI.

SHAP analysis provided deeper insight into how meteorological factors contributed to model predictions [18,19]. The global SHAP summary (Figure 9) identified mean daily temperature, solar radiation, and minimum temperature as the top three most influential features for HRI prediction. Precipitation, wind speed, and humidity had secondary effects, suggesting that direct thermal exposure, rather than transient weather events, predominantly drives HRI risk.

**Figure 10:**
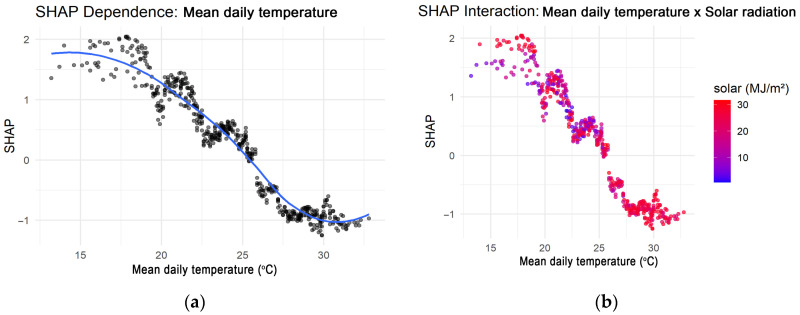
(**a**) SHAP dependence plot for mean daily temperature, highlighting a nonlinear increase in HRI risk above approximately 25 °C. (**b**) SHAP interaction plot between mean daily temperature and solar radiation illustrating their synergistic effect on HRI risk.

The SHAP dependence plot for mean daily temperature (Figure 10a) displayed a clear monotonic trend, with higher temperatures progressively increasing predicted HRI risk. The steepest rise occurred between ~20 °C and 25 °C, suggesting a threshold where small temperature increments produce disproportionately higher predicted risk. This pattern aligns with physiological heat-stress responses and supports an empirically defined temperature threshold for HRI onset in temperate urban environments. Moreover, interaction analysis further uncovered synergistic effects between mean daily temperature and solar radiation (Figure 10b) [18,19]. At higher solar radiation levels (>20 MJ/m^2^), the marginal effect of temperature on SHAP values increased substantially, indicating a multiplicative impact of combined heat and solar exposure. This interaction underscores the importance of considering composite thermal indices rather than single-variable thresholds in operational warning systems.

**Figure 11:**
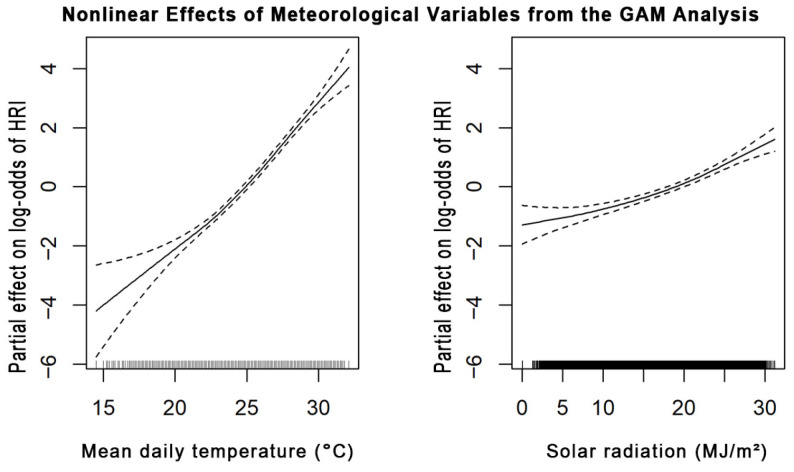
GAM smooth curves for mean daily temperature (**left**) and solar radiation (**right**).

To statistically validate the nonlinear risk patterns observed by SHAP, GAMs were employed (Figure 11). Mean daily temperature and solar radiation displayed monotonic, nonlinear increases in estimated log-odds of HRI occurrence, confirming that heat exposure progressively amplifies risk. The first derivative of the GAM smooth function identified an inflection point at 27.4 °C, where the slope of the log-odds curve reached its maximum. The derivative began to increase around 22–24 °C, defining the onset of the transition zone leading to a rapid escalation in HRI risk. This threshold was derived through a statistical approach rather than visual inspection, thereby providing a quantitative validation of the SHAP-identified transition zone (~25 °C). Methodologically, integrating SHAP and GAM combines interpretability with statistical validation, ensuring that the identified thresholds represent genuine, reproducible risk boundaries.

Collectively, these calibration and explainability analyses confirm that the XGBoost (ROSE) model delivers robust predictions while offering transparent, interpretable, and policy-relevant insights. The identified nonlinear and interaction effects among meteorological variables reinforce the development of adaptive, data-driven early warning frameworks tailored to local climatic conditions.

## 5. Discussion

This study developed an explainable ML model to predict daily HRI occurrence across seven South Korean metropolitan cities using meteorological data. The XGBoost based framework demonstrated strong discriminative and calibration performance (AUC = 0.895, accuracy = 84.3%, Brier score = 0.1261) and provided transparent interpretability through SHAP and GAM analyses. By integrating model performance, calibration, and explainability, this research offers both methodological and practical contributions to climate-sensitive health forecasting.

### 5.1. Interpretation of Key Findings

The XGBoost model attained a robust balance between sensitivity and specificity using ROSE-based class rebalancing. This improvement highlights the value of addressing data imbalance when predicting rare but clinically significant events such as HRI. Calibration and decision curve analyses confirmed well-calibrated probability estimates and substantial net benefit across operational thresholds, validating its suitability for early warning applications [6,7,8,12,13,14,15,16,17].

The explainability results identified mean daily temperature, solar radiation, and minimum temperature as the most influential predictors, confirming that sustained heat exposure and limited nocturnal cooling are key HRI risk factors. SHAP dependence plots revealed a threshold ~24–25 °C, above which the predicted HRI risk increased sharply, corresponding to the onset of physiological heat stress and impaired thermoregulation. Moreover, interaction analysis showed that high solar radiation (>20 MJ/m^2^) significantly amplified temperature-related risk, demonstrating a synergistic effect between ambient temperature and radiative load. These findings align with prior epidemiological research of nonlinear heat-health relationships and synergistic impacts of temperature and solar exposure on morbidity and mortality [5,8,13].

### 5.2. Implications for Public Health and Policy

Current heat wave alert systems in South Korea primarily rely on temperature thresholds and simple indices [8,9,13]. Our findings indicate that explainable ML models can better capture complex, region-specific environmental risk patterns, providing an empirical foundation for adaptive warning criteria. For instance, regions such as Seoul and Busan, which recorded higher HRI incidence despite comparable mean temperatures, could benefit from locally calibrated alert thresholds based on the combined effects of temperature, solar radiation, and humidity.

SHAP-based visualizations also serve as communication tools to improve public understanding of risk by identifying the weather conditions that most strongly influence daily HRI probability. The interpretability of the proposed framework facilitates its integration into operational early warning systems, assists policymakers in prioritizing resources, issues city-level alerts, and tailors public advisories to high-risk conditions [18,19,26]. Overall, the model bridges data science and public health practice by translating complex meteorological information into transparent, actionable insights.

### 5.3. Contribution to the Field

Unlike conventional regression or threshold-based approaches, this study leverages SHAP-derived feature contributions to move beyond average effects. Visualizing variable importance, nonlinear responses, and interactions helps bridge the gap between ML models and actionable guidance for decision-makers. The approach aligns with the P4 medicine paradigm: Predictive, Preventive, Personalized, and Participatory, by emphasizing anticipatory prediction, interpretability, and public engagement [27]. Furthermore, combining SHAP and GAM ensures both statistical validation and visual transparency, setting a framework for future climate-health modeling research.

### 5.4. Limitations and Future Directions

Several limitations of this study are as follows. First, this study incorporated only meteorological predictors; individual-level demographic, socioeconomic, and behavioral variables (e.g., age, occupation, housing, comorbidities) influencing heat vulnerability were unavailable.

Second, although temporal validation using 2025 data confirmed generalizability under unseen conditions, the five-fold random cross-validation applied during training may not fully preserve temporal dependency among observations. Because the primary objective of this study was to evaluate overall model generalizability rather than short-term forecasting, random cross-validation was adopted to ensure a stable and balanced distribution of heat events across folds. Nevertheless, we acknowledge this methodological limitation and suggest that future studies employ blocked or rolling-window cross-validation to further account for temporal structure and seasonality.

Third, while class rebalancing improved performance, additional enhancements, such as ensemble stacking or cost-sensitive learning optimization, could further enhance recall in extremely rare-event settings [12,13,14,15,16,17].

Future research should integrate real-time meteorological APIs, health surveillance data, and emergency response data to establish an automated early detection and intervention pipeline [27,28]. Moreover, linking environmental predictions with hospital admissions and emergency transport data would strengthen the model’s real-world applicability and enable proactive, data-driven public health responses to extreme heat events.

Recent research has highlighted that the health impacts of extreme heat extend beyond traditional non-infectious HRIs to include infectious diseases as well [29]. High temperatures have been shown to accelerate the proliferation and geographic expansion of vectors such as mosquitoes and ticks, and to influence water quality and host susceptibility, collectively increasing the risk of heat-sensitive infectious diseases such as dengue fever and West Nile virus [29]. In this broader context, the ML framework proposed in this study could be expanded by incorporating vector ecology, human mobility data, and environmental or water-quality indicators to enable early detection of heat-induced infectious disease risks. Moreover, advances in graph-based contact tracing models and large-scale healthcare data analytics have demonstrated the potential of AI for early warning and outbreak mitigation in infectious disease settings [30,31]. Integrating such approaches with the present model may facilitate the development of a unified early warning system that captures both non-infectious and infectious heat-related health risks.

## 6. Conclusions

This study developed an XGBoost-based framework to predict HRI using meteorological data collected from 2021 to 2025 across seven South Korean metropolitan cities. The model achieved strong discriminative performance (AUC = 0.895, accuracy = 84.3%) in temporal validation with 2025 data and provided robust, interpretable predictions through XAI techniques, particularly SHAP analysis [10,11,20,21]. The explainability results determined average temperature and solar radiation as the most influential predictors, visualizing their nonlinear and interactive effects on HRI risk [18,19].

SHAP-based analysis identified a critical threshold of ~24–25 °C, above which HRI risk increased sharply, with high solar radiation further amplifying temperature-related risk. These findings underscore the need for multidimensional indicators over simple temperature thresholds for public health alert systems. By illustrating how specific environmental factors drive model predictions, this approach moves beyond conventional statistical interpretation and enhances transparency and trust in AI-assisted decision-making.

The proposed framework demonstrates practical applicability for national and regional heat-health surveillance systems. It could complement the KDCA’s HRI monitoring network by enabling real-time risk assessment and proactive interventions during extreme heat events [3,7,22,23]. At the regional level, it can support city-specific early warning systems that incorporate temperature, solar radiation, and humidity to trigger timely alerts. Model outputs could also inform resource allocation and risk communication, such as the deployment of emergency personnel, operation of cooling centers, and targeted outreach to vulnerable populations, including the elderly, outdoor workers, and individuals with chronic conditions.

To further improve generalizability and operational readiness, future research should validate the framework across diverse climatic regions and incorporate sociodemographic and behavioral variables influencing heat vulnerability. Integration with health surveillance databases, wearable sensors, and environmental monitoring networks could further enhance the model’s precision, scalability, and real-time utility.

Furthermore, recent evidence suggests that heat exposure may indirectly increase the burden of infectious diseases by influencing vector ecology, water quality, and host susceptibility [29]. Accordingly, the XAI-based prediction model developed in this study could be extended to integrate meteorological, environmental, and epidemiological inputs for the early detection of heat-sensitive infectious diseases, as demonstrated in recent advances in graph-based contact tracing algorithms and large-scale healthcare data analytics [30,31]. Such an expansion would support a more comprehensive early warning architecture capable of addressing both non-infectious and infectious heat-related health risks in a warming climate.

Consequently, combining ML with XAI yields interpretable, accurate, and actionable HRI prediction models aligned with the P4 medicine principles: Predictive, Preventive, Personalized, and Participatory [27]. Embedding such models within existing public health infrastructure can support policymakers in strengthening adaptive resilience and protecting population health amid escalating heat risks in a changing climate.

## Figures and Tables

**Table 1:** Clinical manifestations of HRIs.

Condition	Clinical Features	Severity
Heat cramps	Painful muscle spasms, usually in legs or abdomen	Mild
Heat exhaustion	Heavy sweating, weakness, nausea, dizziness, headache	Moderate
Heat syncope	Sudden dizziness or fainting, usually from prolonged standing	Moderate
Heatstroke	High body temperature (>40 °C), confusion, unconsciousness, seizure	Severe, life-threatening

**Table 2:** Meteorological predictor variables used in model training and evaluation.

Variable	Unit	Definition
Mean daily temperature	°C	Arithmetic mean of hourly air temperatures over a day
Maximum temperature	°C	Highest air temperature recorded within a day
Minimum temperature	°C	Lowest air temperature recorded within a day
Temperature range	°C	Difference between daily maximum and minimum temperatures
Mean daily relative humidity	%	Mean of hourly relative-humidity observations over a day
Minimum relative humidity	%	Lowest hourly relative humidity recorded within a day
Precipitation	mm	Total daily accumulated rainfall
Mean daily wind speed	m/s	Mean wind speed measured over a day
Solar radiation	MJ/m^2^	Total solar energy received per unit area per day

Each day generally refers to a 24 h observation period, from 00:00 to 23:59 Korea Standard Time(KST) managed under the KMA Guidelines for the Quality Control. For precipitation, however, daily totals are defined as observations from 09:00 KST on the current day to 09:00 KST on the following day, or, in some cases for precipitation, from 21:00 KST on the previous day to 21:00 KST on the current day, as specified in the KMA Quality Control Guidelines [3].

**Table 3:** Class distribution of the training and test datasets, before and after class rebalancing.

Dataset	Positive (HRI = 1)	Negative (HRI = 0)	Positive Rate (%)	Description
Training set	990	2762	26.3%	Natural distribution
Test set	223	323	40.8%	Temporal test set
Training set (weighted)	990	2762	26.3%	Cost-sensitive adjustment
Training set (ROSE)	1865	1887	49.7%	Synthetic oversampling

**Table 4:** City-level descriptive statistics of major meteorological and intercity comparison results.

Variable	Mean Temperature ^1^ (°C)	Relative Humidity (%)	Solar Radiation (MJ/m^2^)
Gwangju	24.97 ± 3.00	80.85 ± 12.36	18.25 ± 7.40
Daegu	25.09 ± 3.44	70.73 ± 12.27	18.21 ± 7.21
Daejeon	24.75 ± 3.34	73.37 ± 11.80	17.99 ± 7.45
Busan	24.42 ± 3.25	76.69 ± 10.54	18.86 ± 8.27
Seoul	24.89 ± 3.46	72.51 ± 11.19	16.87 ± 7.80
Ulsan	24.01 ± 3.37	77.17 ± 11.09	18.53 ± 5.87
Incheon	23.90 ± 3.53	75.16 ± 11.81	18.00 ± 7.73
*p*-value ^2^	<0.001	<0.001	0.0002

^1^ Mean ± SD. ^2^ Significant between-city differences were observed for all three variables (ANOVA, *p* < 0.001), indicating that despite similar overall ranges, the mean levels of temperature, humidity, and solar radiation varied significantly among the seven metropolitan areas.

**Table 5:** City-level descriptive statistics of daily HRI cases.

Variable	Mean ± SD	Median (IQR)	Max	Population (×10^6^) ^1^	Mean Daily Rate ^2^
Gwangju	0.38 ± 0.92	0 (0–0)	6	1.40	0.0271
Daegu	0.46 ± 1.03	0 (0–0)	7	2.36	0.0195
Daejeon	0.32 ± 0.75	0 (0–0)	7	1.44	0.0222
Busan	0.69 ± 1.53	0 (0–1)	12	3.25	0.0212
Seoul	1.55 ± 3.24	0 (0–2)	27	9.32	0.0166
Ulsan	0.56 ± 1.29	0 (0–1)	12	1.09	0.0514
Incheon	1.11 ± 2.71	0 (0–1)	43	3.04	0.0365
*p*-value ^3^					0.423

Each city included 536 observation days (May–September 2021–2024). ^1^ Population data were obtained from the Resident Registration Statistics published by the Ministry of the Interior and Safety (MOIS), Republic of Korea, as of August 2025. ^2^ Mean daily incidence rate of HRI per 100,000 population. ^3^ Intercity comparisons based on population-adjusted HRI incidence were assessed using the Kruskal–Wallis test, which indicated no statistically significant differences across cities (*p* = 0.423).

**Table 6:** Performance metrics of classification models for predicting HRI occurrence.

Model	AUC	Accuracy	Sensitivity	Specificity	Precision	F1-Score
Logistic ^1^	0.863	0.827	0.583	0.915	0.711	0.641
RFs ^2^	0.854	0.823	0.574	0.912	0.701	0.631
SVM ^3^	0.827	0.819	0.494	0.936	0.734	0.591
k-NN ^4^	0.824	0.804	0.529	0.903	0.661	0.588
XGBoost ^5^	0.860	0.820	0.572	0.909	0.692	0.626
XGBoost (Weighted)	0.857	0.807	0.318	0.9593	0.770	0.512
XGBoost (ROSE ^6^)	0.853	0.778	0.788	0.768	0.771	0.779

The upper five models were trained on the natural class distribution. The lower two XGBoost variants applied class-rebalancing strategies: cost-sensitive weighting (*scale_pos_weight* = 2.8) and synthetic oversampling via the ROSE algorithm. Cross-validation metrics were obtained on the 2021–2024 training period, ^1^ Logistic: Logistic Regression; ^2^ RFs: Random forest; ^3^ SVM: support vector machine; ^4^ k-NN: k-nearest neighbors; ^5^ XGBoost: eXtreme Gradient Boosting; ^6^ ROSE: Random Over-Sampling Examples.

## Data Availability

https://www.kdca.go.kr/board/board.es?mid=a20205030102&bid=0004&&cg_code=C01, https://data.kma.go.kr/climate/RankState/selectRankStatisticsDivisionList.do?pgmNo=179, accessed on 31 October 2025.

## Abbreviations

The following abbreviations are used in this manuscript:

HRI	Heat-related illnesses
HI	Heat index
XAI	Explainable artificial intelligence
ML	Machine learning
RFs	Random forests
SVM	Support vector machine
k-NN	k-nearest neighbors
XGBoost	Extreme gradient boosting
SHAP	Shapley additive explanations
ROSE	Random over-sampling examples
ROC	Receiver operating characteristic
AUC	Area under the curve
